# Cancer Resource and Information Support (CRIS) for Bladder Cancer Survivors and Their Caregivers: Development and Usability Testing Study

**DOI:** 10.2196/41876

**Published:** 2023-06-22

**Authors:** Michael A Diefenbach, Allison Marziliano, Elizabeth J Siembida, Thomas Mistretta, Halie Pfister, Andrea Yacoub, Kelli Aibel, Priya Patel, Emmanuel Lapitan, Erin K Tagai, Marc Smaldone, Suzanne M Miller

**Affiliations:** 1 Institute of Health System Science Feinstein Institutes for Medical Research Northwell Health New York, NY United States; 2 Cancer Prevention and Control Fox Chase Cancer Center Temple University Health System Philadelphia, PA United States; 3 Surgical Oncology Fox Chase Cancer Center Temple University Health System Philadelphia, PA United States

**Keywords:** muscle invasive bladder cancer, behavioral intervention development, ORBIT model, usability testing, web-based intervention

## Abstract

**Background:**

Bladder cancer survivors and their caregivers face profound practical (eg, use of stoma appliances and care for urinary diversion methods) and psychosocial (eg, depression and anxiety) challenges after surgical treatment with cystectomy.

**Objective:**

To improve the health-related quality of life and postsurgical outcomes of both bladder cancer survivors and their caregivers, the team, in collaboration with Sourcetop, Inc (software design) and Dappersmith (graphic design), developed the Cancer Resource and Information Support (CRIS) software. The purpose of this manuscript is to report on the development and usability testing of the CRIS software.

**Methods:**

The development of the CRIS software was guided by the Obesity-Related Behavioral Intervention Trials (ORBIT) model for developing behavioral treatments for chronic diseases. The ORBIT model is unique in that it proposes a flexible and progressive process with prespecific clinically significant milestones for forward movement and returns to earlier stages for refinement, and it facilitates communication among diverse groups by using terminology from the drug development model. This paper focuses on 2 phases of the ORBIT model: phase IA: define and IB: refine. During phase IA, the study team developed solutions for the stated clinical problem—adjustment to life post cystectomy—by reviewing the literature and collecting feedback from clinicians, professional organizations, bladder cancer survivors, and their caregivers. During Phase IB, the study team focused on tailoring content in the CRIS software to the user as well as usability testing with 7 participants.

**Results:**

The finished product is CRIS, a web-based software for survivors of bladder cancer and their caregivers to serve as a health management and lifestyle resource after surgery. Overarching themes from phase IA (participant feedback) included how to use new medical equipment, tips and tricks for easier living with new medical equipment, questions about health maintenance, and questions about lifestyle modifications. To accommodate our target population, we also incorporated recommendations from the Americans with Disabilities Act for website design, such as large text size, large paragraph spacing, highly contrasting text and background colors, use of headings and labels to describe the purpose of the content, portrait orientation without the need for horizontal scrolling, multiple ways to access a web page within a set of pages, ability to navigate web pages in sequential order, and in-text links that are descriptive. Usability participants evaluated CRIS very positively, indicating that it was easy to use, the functions were well-integrated, and if available, they would use CRIS frequently.

**Conclusions:**

CRIS, developed over the course of 18 months by integrating feedback from experts, literature reviews, and usability testing, is the first web-based software developed for bladder cancer survivors and their caregivers to help them adjust to life following cystectomy. The efficacy of CRIS in improving patients’ and caregivers’ quality of life is currently being evaluated in a randomized controlled trial.

## Introduction

Bladder cancer is the most common malignancy of the urinary system that affects both men and women, primarily over the age of 55 years. It is estimated that there will be approximately 82,290 new cases of bladder cancer and 16,710 deaths from bladder cancer in 2023 in the United States alone [1]. About half of the bladder tumors are invasive, reaching the muscle and fat layers of the bladder wall or spreading to nearby tissues [1]. Recommended treatment for muscle invasive bladder cancer consists of neoadjuvant chemotherapy and subsequent cystectomy, or removal of the urinary bladder [2]. Following cystectomy, surgeons reconstruct one of three types of urinary diversion methods: (1) an ileal conduit, which involves a stoma for an external pouch to collect urine; (2) an Indiana pouch, which involves a stoma and an internal pouch requiring survivors to self-catheterize; or (3) a neobladder, a bladder reconstructed with intestinal tissue in the place of the removed one. Each of these urinary diversion types results in a long surgical recovery and permanent, long-term lifestyle changes for survivors and their caregivers.

It is no surprise, then, that bladder cancer survivors and their caregivers face profound practical and psychosocial challenges after surgical treatment with cystectomy [3-7]. Practical challenges include the use of stoma appliances and caring for their urinary diversion method. Survivors also experience reductions in emotional well-being, increased posttreatment depression and anxiety, and restrictions in activities of daily living, including physical and sexual activity [8-10]. Such adjustment difficulties might lead to a long-term loss of independence and delay the return to a new normal life [4,5,8]. Caregivers often have to adjust to an expanded support role while negotiating their regular family and professional obligations; increased worry about the survivor and heightened levels of distress have also been mentioned [11]. Therefore, there is an urgent need for an intervention to address the practical and psychosocial challenges associated with life post cystectomy.

Existing support has been ineffective in preparing patients and caregivers for these practical and psychological challenges. To facilitate the adjustment to life post surgery, survivors typically receive industry-branded pamphlets or brochures, which are of varying quality and usefulness. Rarely do stoma nurses spend time providing in-depth care instructions to survivors before discharge from the hospital. When these conversations do occur, their timing can be problematic, as caregivers are often not present during these discussions. The discharge process often involves many other tasks and may be complicated by pain medication–induced confusion and disorientation [4,5]. Consequently, discharge is not an optimal time for teaching new medical management techniques. As a result, survivors and caregivers are often unprepared for the postsurgical challenges and adjustments when arriving home, contributing to poor psychosocial functioning [5,8,12].

In light of this, software and web-based learning offer a promising alternative and have shown to be effective as patient and caregiver education tools. There are many advantages to developing and providing software-based solutions. They are as follows: (1) patients can consume the information at their own schedule, in a private place, and as often as they desire; (2) information can be shared with caregivers and family members; (3) information can be transmitted through multiple channels (personal computer, tablet, or phone); (4) content can be augmented through videos, graphics, testimonials, and interactive activities; (5) content can be tailored to specific time-points in the recovery trajectory, treatment approaches, and targeted to specific groups; (6) software-based solutions transcend geographical barriers, and can easily be updated; and (7) software can be used to collect data on patient recovery progress and trajectory, allowing for immediate feedback, which might provide the motivation to continue with recommended self-care actions. Overall, software-based patient education is effective in increasing disease-related knowledge and improving health behaviors and self-management skills [13-16]. However, there is no existing research on web-based tools to specifically address the unmet needs of patients and caregivers diagnosed with bladder cancer during the cystectomy process.

To overcome these gaps and improve the health-related quality of life and postsurgical outcomes of both bladder cancer survivors and their caregivers without further taxing the time of health care providers, the team developed the Cancer Resource and Information Support (CRIS) software. CRIS is an interactive web-based program developed for bladder cancer survivors who will undergo surgical treatment and their caregivers. It is unique in its design to be used before and after surgery; it includes caregivers in all intervention components; and all materials can be accessed on demand. CRIS uses videos and graphics to deliver comprehensive educational and psychosocial information about postsurgical care tailored to the diversion type. CRIS is currently being evaluated in a full-scale multisite randomized controlled trial, and this paper describes the development and usability testing of the CRIS software.

## Methods

### Ethics Approval

The study was conducted at Northwell Health and the affiliated Feinstein Institutes for Medical Research in New York and Fox Chase Cancer Center/Temple University Health System in Pennsylvania. All study procedures were approved by the Institutional Review Boards of participating institutions (Northwell Health: 18-0400; Fox Chase Cancer Center: 18-1036). A member of the study team obtained informed consent from all research participants. Only members of the study team who have been approved by the institutional review board have access to the data, which has now been deidentified and is stored on an encrypted and privacy-protected health system server. Participants were provided with a US $100 gift card for study completion.

### Guiding Theory: Obesity-Related Behavioral Intervention Trials (ORBIT) Model

**Figure 1:**
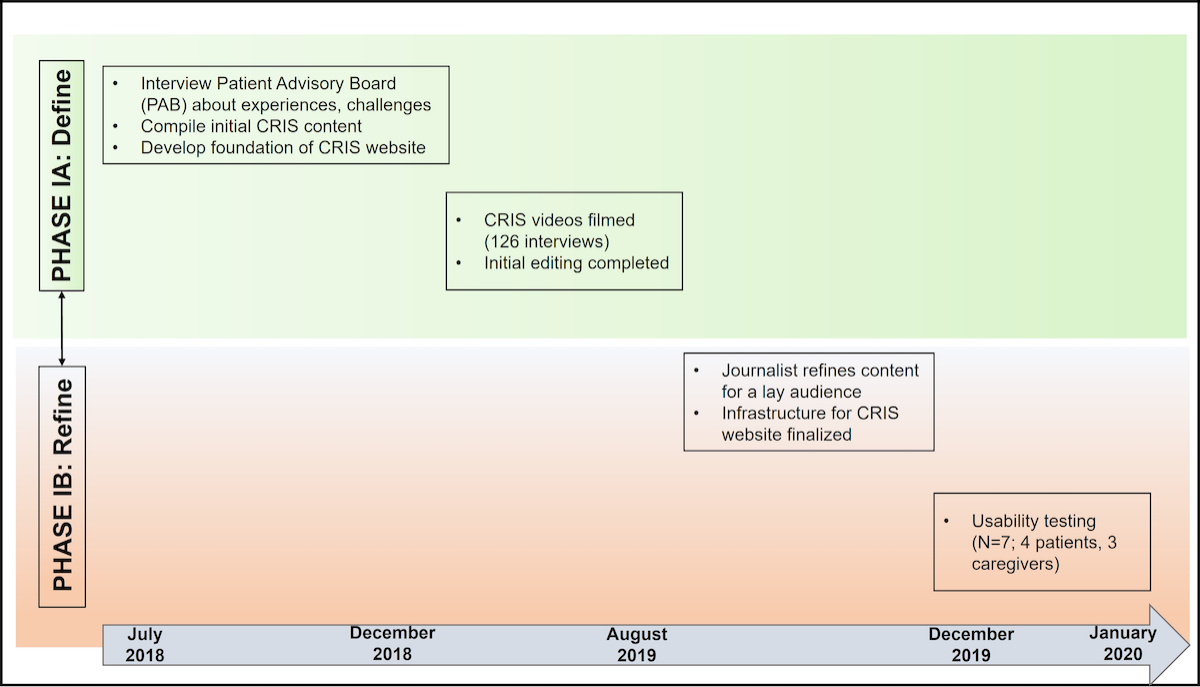
Overview of Obesity-Related Behavioral Intervention Trials (ORBIT) model phases associated with CRIS development steps and timeline. CRIS: Cancer Resource and Information Support.

The development of the CRIS software was guided by the Obesity-Related Behavioral Intervention Trials (ORBIT) [17] model for developing behavioral treatments for chronic diseases. The ORBIT model delineates the development, refinement, and testing processes into various phases. We focus on the first phases (phases IA and IB) of the model in this manuscript. During phase IA, we developed and refined solutions for our stated clinical problem: adjustment to life post cystectomy. During phase IB, we refined the intervention content to best fit the needs of our target population. A key feature of the ORBIT model is the emphasis on the early stages of program intervention development through an iterative process of development, feedback, and refinement sprints. Members of the target audience are an important part of this process, and we integrate their feedback into each phase. Figure 1 shows the ORBIT model integrated with the CRIS development process over the span of 18 months, beginning in July 2018 and ending with the finalized product in January 2020.

### Phase IA: Define the Clinical Problem

#### Identifying the Clinical Problem

In addition to what has been indicated in the literature, our team conducted 16 individual interviews and 1 focus group (N=3) with patients who had received treatment for muscle invasive bladder cancer. During the interviews, we solicited feedback on unmet needs in multiple domains: informational, social, psychological, instrumental, and medical. The team asked patients to categorize when needs were most prevalent during the disease and recovery trajectory (ie, at diagnosis, up to 3 months post-op, and beyond 3 months).

#### Creation of a Patient Advisory Board

A formal patient advisory board (PAB) was formed to clarify the preferences of survivors and caregivers on the content they would like to see on the CRIS website. The PAB consisted of 4 individuals: 3 bladder cancer survivors who underwent cystectomy, each of whom received one of the 3 different surgical procedures for urinary diversion, and a caregiver. We conducted individual interviews with members of the PAB to guide the development of the CRIS website. PAB members reported on their information needs, their preferred mode of intervention delivery, and the type of content that was most important to them.

#### Text and Multimedia Content Development

Recommendations from collaborating clinicians from the 2 institutions and guidelines from professional organizations including the Bladder Cancer Advocacy Network, the American Cancer Society, and the American College of Surgeons served as the basis for the CRIS website’s content. To accommodate our target population and foresee potential user challenges, we also incorporated recommendations from the Americans with Disabilities Act for website design [18]. Some of these recommendations include large text size, large paragraph spacing, highly contrasting text, and background colors, use of headings and labels to describe the purpose of the content, portrait orientation without the need for horizontal scrolling, multiple ways to access a web page within a set of pages, ability to navigate web pages in sequential order, and in-text links that are descriptive.

To make the CRIS website easy to understand, we ensured that content was delivered through multiple approaches (eg, videos and graphics). The PAB suggested specific topics that they would like to see in video format. These topics included physicians speaking about the specific surgical procedure, nurses speaking about stoma care, and other survivors discussing their personal experiences. To address these content requests, the investigative team and PAB curated a number of frequently asked questions about treatment and survivorship needs. We videotaped physicians and stoma nurses from each institution answering these questions. This resulted in 2 versions of the program: a Northwell version with Northwell physicians and nurses and a Fox Chase Cancer Center version, representing Fox Chase physicians and nurses. The 2 versions are of identical content but are intended for the respective survivor populations, with their own physicians and nurses providing their educational information. In total, 126 videos were included in CRIS. These included 81 videos for the participants from Northwell Health and 28 videos for the participants from Fox Chase Cancer Center. These videos were mostly of physicians at various locations speaking about bladder cancer and its treatment. The American College of Surgeons also provided permission to incorporate 5 of their educational videos depicting animated surgical procedures. Lastly, 1 video on how to engage in pelvic exercises was extracted from the internet and included.

The website underwent several rounds of reviewing and editing between the study team, web developers, and clinicians, including urologists and nurse practitioners. All written, graphic, and video information included was vetted and approved by our medical collaborators.

### Phase IB: Refine CRIS’ Content

#### Personalization of Viewing Content

During this phase, we focused on tailoring the information to the users. Survivors and caregivers are given access to information specific to their health system and chosen surgical procedures. For example, a survivor enrolled at Northwell Health who had an ileal conduit will, by default, only sees information pertaining to ileal conduit care presented by Northwell physicians or nurses. This ensures our users are seeing only the information that is pertinent to them. We also designed separate sections of the website for survivors and caregivers so that each can more easily find information pertaining to their needs.

#### Usability Testing

After the first version of the website was completed, we conducted usability testing to explore our prototype CRIS website. Guidelines suggest that the more severe usability problems are most often detected by the first few participants, and more than 80% of usability issues are discovered with just 4-5 users [19-21].

Our sample for usability testing consisted of 7 participants (5 survivors and 2 caregivers). Of this sample, 4 participants were from our PAB. Three of the survivors had an ileal conduit, 1 had an Indiana pouch, and 1 had a neobladder.

Following informed consent, participants were given a tablet or laptop with the prototype website and were asked to “think aloud” as they navigated through the pages. Four of the participants viewed the program on a tablet (a Samsung Galaxy Tab A 8”), and 3 viewed it on a laptop. Participants involved in this phase freely explored CRIS for 15-20 minutes; their narrative thoughts and observations were audio-recorded, and a research assistant took extensive notes. Study staff would note any general remarks that participants made, including any difficulties with the program or elements of the program that the participants liked. Study staff helped participants if necessary. After they were finished exploring, survivors were asked to complete specific tasks to simulate real-life situations and assess how intuitive and user-friendly the website was. Examples of these discreet tasks include “navigate to the home page” and “find and click on the section about traveling with your medical equipment.” At the end of the usability session, participants completed the brief, 10-item System Usability Scale, the industry standard for measuring usability [22]. Questions are answered on a 5-point response scale (strongly disagree to strongly agree). Feedback from the usability testing led to a final round of editing and revisions, which ultimately produced the final CRIS website (see Figure 1).

## Results

### Phase IA Results

**Figure 2:**
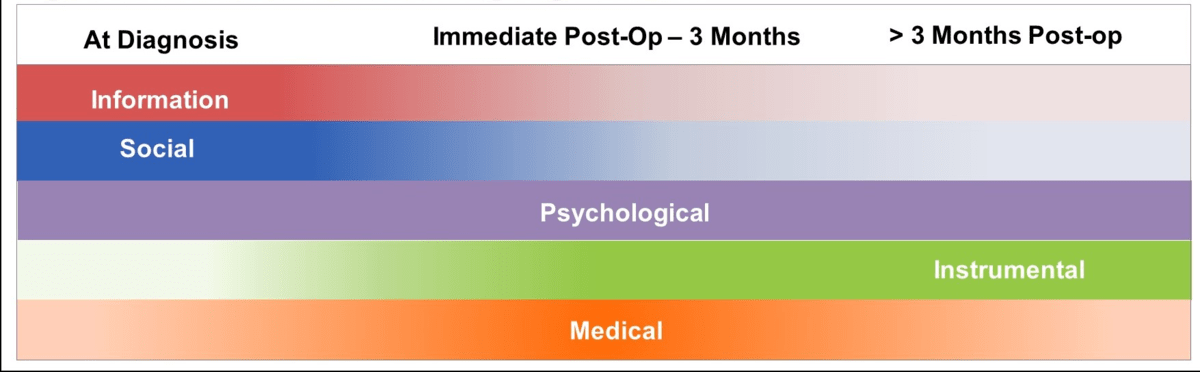
Assessment of bladder cancer patients’ unmet needs across the caregiving continuum. This graphic displays the results of interviews on the unmet needs of patients and how these needs shift over time (indicated by the strength of the color bar), though psychological needs remain constant.

The 19 patients (mean age 66 years) who underwent an initial interview (n=16) or focus group discussion (n=3) to identify the clinical problem reported unmet needs within the informational, social, psychological, instrumental, and medical domains (see Figure 2), thus confirming existing reports (ie, insufficient training on self-care and lack of informational and emotional support). This sample was 84% (n=16) non-Hispanic White, 74% (n=14) had at least a high school diploma or GED, and 47% (n=9) were married. We found that the needs of patients shift over time (as indicated by the strength of the color bar in Figure 2), with the exception of needs in the psychological domain, which appear to be constant. For example, while psychological symptoms (particularly distress, depression, and fear of recurrence) were constant problems for patients, informational and social needs were strongest at the time of diagnosis and became less prevalent over the next 3 months. To illustrate, patients expressed insufficient discussion with health care providers at diagnosis about treatment options, postoperative recovery and self-care, illness trajectory, and financial obligations. Patients also reported a high rate of rehospitalizations due to stoma care complications, which is consistent with the literature [23].

**Figure 3:**
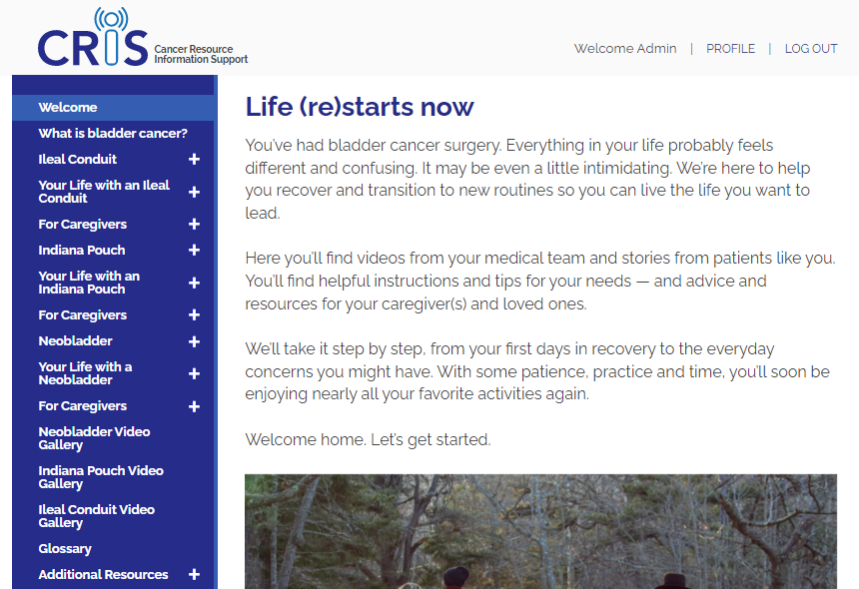
Cancer Resource and Information Support (CRIS) homepage for an ileal conduit user. The navigation pane on the left-hand side shows the main topics. Subtopics can be viewed after clicking the “+” symbol.

**Figure 4:**
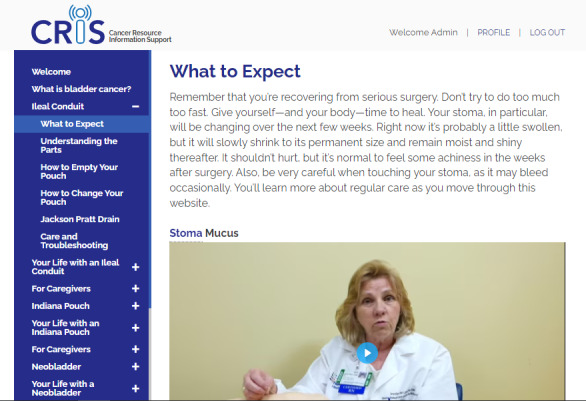
Cancer Resource and Information Support (CRIS) sample page for an ileal conduit survivor. This is a sample page for an ileal conduit user that also shows how the videos are embedded within the relevant pages.

**Figure 5:**
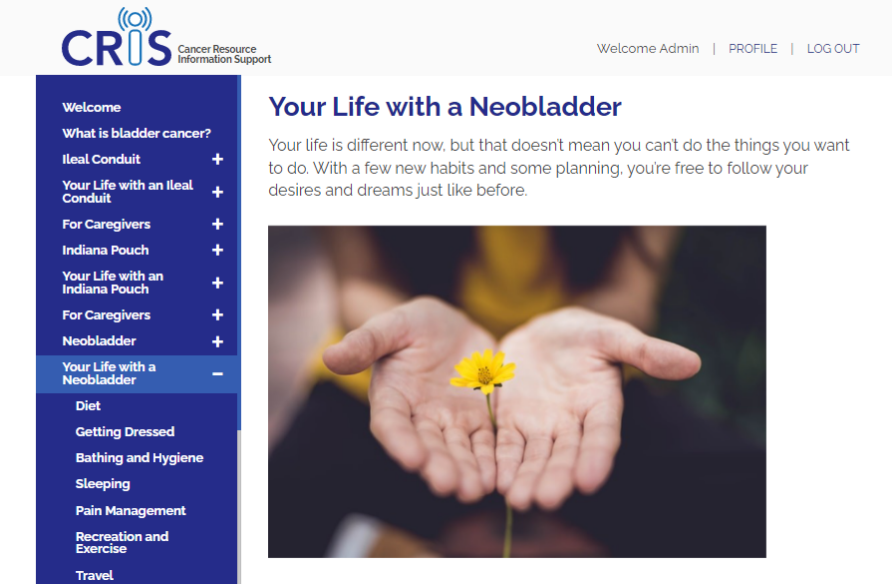
Cancer Resource and Information Support (CRIS) sample page showing the Lifestyle section. This page displays the numerous lifestyle topics that were noted in interviews with our Patient Advisory Board (PAB).

**Figure 6:**
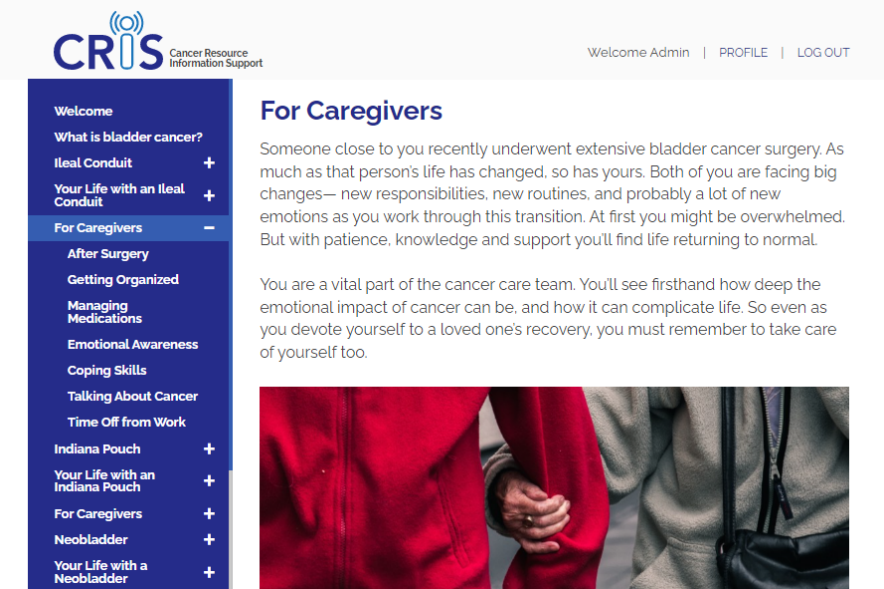
Cancer Resource and Information Support (CRIS) sample page showing the Caregiver section. This page shows the topics specifically for caregivers.

In addition to these initial interviews, our interviews with the formal PAB confirmed our prior observations that survivors of bladder cancer need a resource for health management and lifestyle advice after surgery. Overarching themes included the use of new medical equipment (eg, the type of medical appliances available and their fit on the body, as well as maintenance, cleaning, and reordering of supplies) and other specific tips and tricks that make living with such appliances easier. Other themes that emerged involved questions about health maintenance (eg, hygiene, diet, bathing, sleeping, pain management, and sexual activity) as well as questions about lifestyle modifications (eg, dress, exercise, work, and travel). Caregivers were particularly interested in having a resource to help with managing the care for their loved one, as well as information on emotional support and issues around self-care. Figures 3-6 show sample pages from the CRIS website.

### Phase IB Results

During usability testing, participants reported having little difficulty navigating between pages or seeking specific information. When participants were asked to redirect to specific information on the site, they were able to do so without help from the study team. Overall, participants felt that the layout and presentation of the material were easy to read and follow. They appreciated the way things were worded and the images that were used. One of the caregivers said:

It’s a soothing site, with the colors and videos. The [caregiver] videos are very comforting. [The website] is definitely needed.

Four of the 5 survivors remarked that the website would have been a useful resource before having surgery or during the recovery period immediately after surgery. Participant 1 said:

I think if you’re in the hospital post surgery, I think it’s a great tool. I had my tablet and I was doing a little “Googling,” but I [couldn’t find] a spot where [the information] all was.

Participant 2 also agreed with this sentiment and added, “Even while in rehab, I would have loved to have this.” Some of their feedback, like a search bar, a page titled “talking about cancer,” and “about this website,” was added to the web page to create an easier experience for future users.

Participants mentioned that some of the information on CRIS covered topics was not discussed by their physician and care team, and thus, provided an important additional source of information. This included specifics such as pain medications, being intimate, feelings of guilt, family and medical leave, and the emotional toll of the procedure and recovery process. Participant 4 stated, “I don’t remember anyone coming to tell me about what (I) might experience emotionally.” Some of the participants also gave specific feedback on areas for improvement. Participant 3 noted that some of the images of pouch systems were outdated and that some of the languages regarding a description of the pouch system could be clearer.

At the end of the usability testing, all participants (N=7) completed the System Usability Scale (see Table 1). Overall, survivors evaluated CRIS very positively, indicating that they would use it frequently if it were available, that it was easy to use, and that the functions were well integrated. They also indicated that CRIS was easy to learn and that they felt confident using it.

**Table 1:** Results from System Usability Scale.

Question	System Usability Scale (N=7)		
	Min	Max	Mean (SD)
1. I think that I would like to use this tool frequently	3	5	4.43 (0.976)
2. I found the tool unnecessarily complex.	1	3	1.43 (0.789)
3. I think the tool will be easy to use.	4	5	4.57 (0.534)
4. I think I would need the support of a technical person to be able to use this tool.	1	1	1 (0.000)
5. I found the various functions of this tool were well integrated.	4	5	4.71 (0.488)
6. I thought there was too much inconsistency in this tool.	1	1	1 (0.000)
7. I would imagine that most people would learn to use this tool very quickly.	5	5	5 (0.000)
8. I found the tool’s workflow very cumbersome to use.	1	2	1.14 (0.378)
9. I would feel very confident using the tool.	4	5	4.86 (0.378)
10. I needed to learn a lot of things before I could get going with this system.	1	5	1.71 (1.50)

## Discussion

### Principal Findings

Existing web-based tools for bladder cancer survivors are prognostic tools that serve to facilitate treatment decision-making [24-26]. CRIS is unique because it is the first web-based software developed for bladder cancer survivors and their caregivers to assist in their adjustment to life following cystectomy. Our multidisciplinary team followed an iterative and theory-based approach using the ORBIT model to create the CRIS software. With input from stakeholders and providers, CRIS’ intuitive and user-friendly design can easily be adapted to other cancer types and other chronic diseases characterized by significant posttreatment challenges.

We undertook the development of CRIS because we identified an urgent need to improve survivors’ and caregivers’ knowledge and skills for postcystectomy care. In addition to ensuring that the content on CRIS is relevant to survivors and caregivers, we also designed CRIS with many features specifically for the purpose of increasing user engagement. Currently, engagement with web-based informational resources rests at a suboptimal level of approximately 40%-50% [27]. The engagement features included in CRIS are (1) providing content that was deemed most relevant and necessary as indicated by our PAB, (2) tailoring the content to meet the needs of the specific survivor or caregiver who is viewing the material, and (3) ensuring that the content is easy to navigate and understand.

Web-based programs, such as CRIS, have the potential to transform the survivorship experience as they transcend many of the barriers associated with accessing traditional educational interventions. One commonly cited barrier is clinicians’ limited time [27]. Through CRIS, survivors and caregivers have access to the critical information they need during the entire span of survivorship care, at any time, and from any location. Survivors and caregivers need not wait for clinicians to return their calls, worry about forgetting to ask a specific question when they are speaking to their clinicians, or visit the emergency room for help with their stoma appliances. Clinicians’ and nurse practitioners’ time will be better managed and can be devoted to clinical tasks, such as providing care to those still in the hospital immediately post surgery. CRIS also decreases the perceived or actual stigma associated with asking for practical or emotional support.

### Limitations

There are some limitations to note in relation to the development of CRIS. First, our sample size for the usability testing is limited; however, we did include survivors who engaged in all possible treatment options. Second, at this time, we only have the resources to develop CRIS in the English language; therefore, we were limited to obtaining feedback from English-speaking participants on the development and usability of the program. Third, some of the same participants were used in the initial define phase and again in the usability or refine phase, giving these individuals knowledge about the tool that could put them at an advantage during usability testing, affecting usability outcomes. However, the participants who only participated in the usability phase had the same feedback as those who participated in both phases.

### Future Research Directions

The next steps in this program of research include an evaluation of the efficacy of CRIS in improving quality of life in survivors of bladder cancer and their caregivers through a 2-site randomized controlled trial. The trial will compare CRIS to a time and attention comparison condition that incorporates a standard of care discharge instructions and modules focusing on wellness. While both patients’ and caregivers’ quality of life is the primary outcome, secondary outcomes are fewer infections and nurse-emergency room visits for patients randomized into CRIS. We will conduct moderator (ie, age, gender, and surgical diversion type) and mediator (ie, patient activation and distress) analyses of intervention efficacy, as well as examine the costs and potential savings associated with developing and implementing the CRIS intervention.

### Conclusions

The development of CRIS provides an example of best practices for creating web-based tools. Our use of the ORBIT model and engagement with the target population throughout the development process represent necessary steps to creating a usable, survivor- and caregiver-centered tool. This paper also represents a critical contribution to the bladder cancer survivorship literature, as CRIS will provide both practical and psychosocial support to survivors and their caregivers at multiple points throughout the survivorship trajectory.

## Abbreviations

CRIS: Cancer Resource and Information Support
ORBIT: Obesity-Related Behavioral Intervention Trials
PAB: patient advisory board

## Data Availability

The data sets generated during or analyzed during this study are available from the corresponding author upon reasonable request.

## References

[1] (2023). Key statistics for bladder cancer. American Cancer Society.

[2] Motterle G, Karnes RJ (2020). Timing of treatment for muscle-invasive bladder cancer in the neo-adjuvant chemotherapy era. AME Med J.

[3] Porter MP, Wei JT, Penson DF (2005). Quality of life issues in bladder cancer patients following cystectomy and urinary diversion. Urol Clin North Am.

[4] Somani BK, Gimlin D, Fayers P, N'dow J (2009). Quality of life and body image for bladder cancer patients undergoing radical cystectomy and urinary diversion—a prospective cohort study with a systematic review of literature. Urology.

[5] Mohamed N, Diefenbach M, Goltz H, Lee CT, Latini D, Kowalkowski M, Philips C, Hassan W, Hall SJ (2012). Muscle invasive bladder cancer: from diagnosis to survivorship. Adv Urol.

[6] Stein JP, Penson DF, Lee C, Cai J, Miranda G, Skinner DG (2009). Long-term oncological outcomes in women undergoing radical cystectomy and orthotopic diversion for bladder cancer. J Urol.

[7] Rammant E, Van Hecke A, Decaestecker K, Albersen M, Joniau S, Everaerts W, Jansen F, Mohamed NE, Colman R, Van Hemelrijck M, Fonteyne V (2022). Supportive care needs and utilization of bladder cancer patients undergoing radical cystectomy: a longitudinal study. Psychooncology.

[8] McMullen CK, Kwan ML, Colwell JC, Munneke JR, Davis JV, Firemark A, Brooks N, Grant M, Gilbert SM, Altschuler A (2019). Recovering from cystectomy: patient perspectives. Bladder Cancer.

[9] Hardt J, Filipas D, Hohenfellner R, Egle U (2000). Quality of life in patients with bladder carcinoma after cystectomy: first results of a prospective study. Qual Life Res.

[10] Shih C, Porter MP (2011). Health-related quality of life after cystectomy and urinary diversion for bladder cancer. Adv Urol.

[11] Mohamed NE, Shah QN, Kata HE, Sfakianos J, Given B (2021). Dealing with the unthinkable: bladder and colorectal cancer patients' and informal caregivers' unmet needs and challenges in life after ostomies. Semin Oncol Nurs.

[12] van Ryn M, Sanders S, Kahn K, van Houtven C, Griffin JM, Martin M, Atienza AA, Phelan S, Finstad D, Rowland J (2011). Objective burden, resources, and other stressors among informal cancer caregivers: a hidden quality issue?. Psychooncology.

[13] Wantland DJ, Portillo CJ, Holzemer WL, Slaughter R, McGhee EM (2004). The effectiveness of web-based vs. non-web-based interventions: a meta-analysis of behavioral change outcomes. J Med Internet Res.

[14] Krebs P, Prochaska JO, Rossi JS (2010). A meta-analysis of computer-tailored interventions for health behavior change. Prev Med.

[15] Samoocha D, Bruinvels DJ, Elbers NA, Anema JR, van der Beek AJ (2010). Effectiveness of web-based interventions on patient empowerment: a systematic review and meta-analysis. J Med Internet Res.

[16] Kim A, Park H, Sarkar IN, Georgiou A, de Azevedo Marques PM (2015). Web-based self-management support interventions for cancer survivors: a systematic review and meta-analyses. MEDINFO 2015: eHealth-enabled Health.

[17] Czajkowski SM, Powell LH, Adler N, Naar-King S, Reynolds KD, Hunter CM, Laraia B, Olster DH, Perna FM, Peterson JC, Epel E, Boyington JE, Charlson ME (2015). From ideas to efficacy: The ORBIT model for developing behavioral treatments for chronic diseases. Health Psychol.

[18] (2022). Guidance on web accessibility and the ADA. US Department of Justice Civil Rights Division.

[19] Virzi RA (2016). Refining the test phase of usability evaluation: how many subjects is enough?. Hum Factors.

[20] Nielsen J, Landauer TK (1993). A mathematical model of the finding of usability problems. CHI '93: Proceedings of the INTERACT '93 and CHI '93 Conference on Human Factors in Computing Systems.

[21] Lewis JR (1994). Sample sizes for usability studies: additional considerations. Hum Factors.

[22] Turner C, Lewis J, Nielsen J, Karwowski W, Raton B (2006). Determining usability test sample size. International Encyclopedia of Ergonomics and Human Factors, Second Edition.

[23] Hu M, Jacobs BL, Montgomery JS, He C, Ye J, Zhang Y, Brathwaite J, Morgan TM, Hafez KS, Weizer AZ, Gilbert SM, Lee CT, Lavieri MS, Helm JE, Hollenbeck BK, Skolarus TA (2014). Sharpening the focus on causes and timing of readmission after radical cystectomy for bladder cancer. Cancer.

[24] Gild P, Rink M, Meyer CP (2017). Online tools for patient counseling in bladder and kidney cancer—ready for prime time?. Transl Androl Urol.

[25] Shariat SF, Margulis V, Lotan Y, Montorsi F, Karakiewicz PI (2008). Nomograms for bladder cancer. Eur Urol.

[26] Dancik GM (2015). An online tool for evaluating diagnostic and prognostic gene expression biomarkers in bladder cancer. BMC Urol.

[27] Owen JE, Bantum EO, Gorlick A, Stanton AL (2015). Engagement with a social networking intervention for cancer-related distress. Ann Behav Med.

